# An ominous ECG

**DOI:** 10.1007/s12471-025-02007-5

**Published:** 2025-12-16

**Authors:** M. Libbrecht, T. De Meyer, M. Boulaksil

**Affiliations:** Department of Cardiology, Hartcentrum Kust, AZ Oostende hospital, Oostende, Belgium

An 85-year-old man presented to our emergency department with recent onset retrosternal pain and progressive dyspnoea. His history included CABG with multiple PCIs thereafter and paroxysmal atrial fibrillation. The pain seemed similar to prior cardiac events. His medications were: flecainide, bisoprolol, ticagrelor, rosuvastatin/ezetimibe, and dutasteride/tamsulosin.

Initial physical examination showed a blood pressure of 82/48 mm Hg, a heart rate of 34 bpm, and normal oxygen saturation. Laboratory results showed acute kidney injury with an eGFR of 43 ml/min (CKD-EPI; 3 months before: 76 ml/min) and no inflammation. There were elevated hs-troponin T levels of 243 ng/L, which rose at re-check (273 ng/L). The ECG on admission is shown in Fig. 1. Subsequently, he was transferred to the Cardiac Care Unit.

**Fig. 1:**
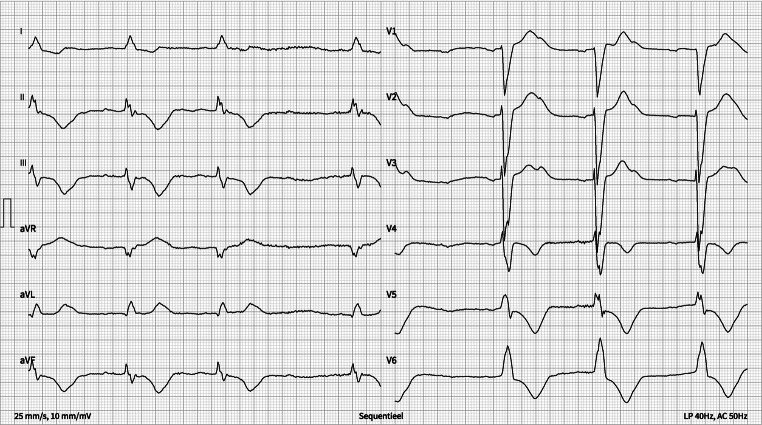
Initial ECG at presentation

What is your (differential) diagnosis?

## Answer

You will find the answer elsewhere in the journal.

